# Editorial: Leaf functional traits: Ecological and evolutionary implications

**DOI:** 10.3389/fpls.2023.1169558

**Published:** 2023-03-21

**Authors:** Karl J. Niklas, Peijian Shi, Johan Gielis, Julian Schrader, Ülo Niinemets

**Affiliations:** ^1^School of Integrative Plant Science, Cornell University, Ithaca, NY, United States; ^2^Bamboo Research Institute, Nanjing Forestry University, Nanjing, China; ^3^Department of Biosciences Engineering, University of Antwerp, Antwerp, Belgium; ^4^School of Natural Sciences, Macquarie University, Sydney, NSW, Australia; ^5^Institute of Agricultural and Environmental Sciences, Estonian University of Life Sciences, Tartu, Estonia; ^6^Estonian Academy of Sciences, Tallinn, Estonia

**Keywords:** elevation, lamina centroid, leaf dry mass per area, leaf shape, leaf size, leaf stoichiometry, plant intraspecific trait variation, urbanization

Foliage provides the primary photosynthetic surfaces of terrestrial vascular plants. Consequently, quantifying and understanding the relationships among the functional traits of leaves are critical to our understanding of terrestrial ecosystem dynamics, nutrient cycles, responses to current global climate change, and the evolutionary trajectories of foliage form and function. Numerous prior studies have investigated relationships among critical leaf functional traits, such as leaf mass, area, shape, nutrient contents, photosynthesis and respiration, stomatal density, stomatal size, leaf vein density, leaf vein length, leaf vein area, areole area (Niklas, 1999; Franks and Farquhar, 2007; Milla and Reich, 2007; Franks and Beerling, 2009; Fiorin et al., 2016; Shi et al., 2021; Shi et al., 2022). However, much remains to be learned, particularly about the functional scaling of leaf characteristics with the traits of other plant organs, with whole plant traits such as plant height and total mass, and how variation in key environmental drivers shape leaf functional traits. The goal of this Research Topic in Frontiers of Plant Science was to bring together a group of ecophysiologists to provide fresh insight into how critical leaf traits respond to environmental variability and what are the implications of environmental-driven variation in leaf traits on plant performance. The Research Topic consists of 15 papers spanning a broad spectrum of research areas ranging from the effects of elevation and water stress on leaf size and shape, urbanization effects, and the influence of a mass extinction event on viable leaf-economic strategies.

## Different perspectives on the allometry of leaf scaling

**Figure 1:**
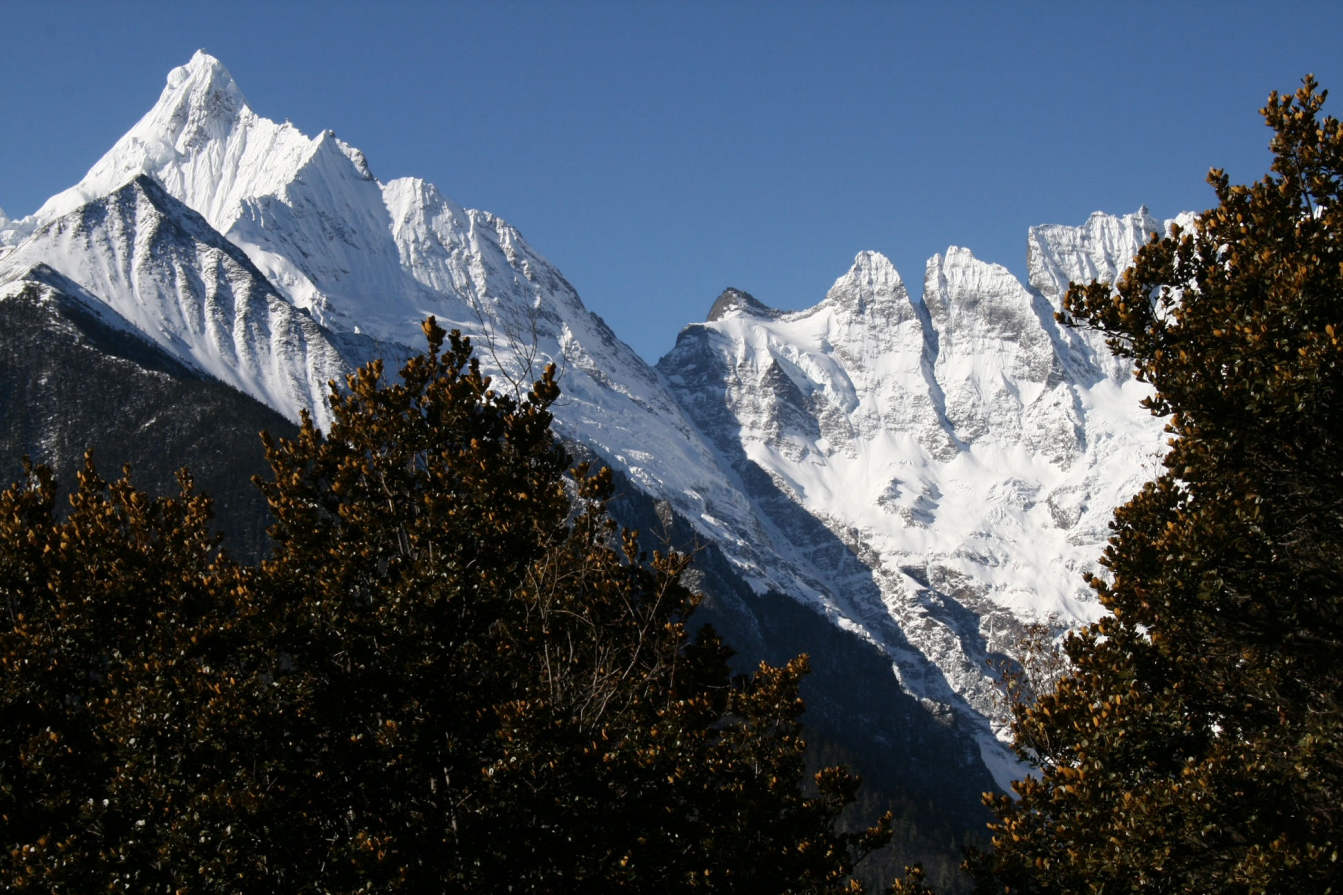
*Quercus pannosa* growing in Shangri-la, Yunnan Province, China (Provided by Dr. Jianzhong Ma). Environmental factors and tree size can significantly affect the leaf functional traits (see Ma et al. in this special issue).

Leaf shape and size play important roles in photosynthetic efficiency of plants through the growing season, and this Research Topic includes four papers looking at fundamental leaf scaling relationships focusing on the effects of plant size, leaf shape, and leaf age that address whether there is disproportionality in mass and area scaling relationships, a phenomenon called “diminishing returns”. Ma et al. studied 60 trees of an alpine evergreen oak (*Quercus pannosa*) to test whether tree size affects leaf shape, size, and leaf dry mass per unit area, and to test whether the proportional relationship between leaf area and the product of leaf length and width is a valid metric for calculating the leaf area of the leaves of trees differing in size (**Figure 1**). They found that tree size significantly influenced leaf shape, size, and leaf dry mass per unit area. Larger trees had larger and broader leaves with lower leaf dry mass per unit area, with lamina centroids closer to the leaf apex than the leaf base. Nevertheless, the proportional relationship between leaf area and the product of leaf length and width was independent of tree size.

To test whether the position of leaf lamina centroid is correlated with the allocation of biomass to the leaf lamina and petiole, Li et al. used a general ovate leaf-shape model to fit the leaf boundary coordinate data of two Lauraceae species, *Cinnamomum camphora* and *Machilus leptophylla*, using >290 leaves for each species. They found that a higher distance from the lamina centroid to the leaf base that connects the petiole and lamina does not necessarily result in a greater investment of mass to the petiole relative to lamina. In fact, the petiole/lamina mass ratio depends on the characteristics of the petiole (which is either short and thick or long and thin).

“Diminishing returns” in leaf economics occurs when lamina mass disproportionately increases with increasing leaf surface area (Niklas et al., 2007). Guo et al. tested whether both leaf fresh and dry mass manifest diminishing returns using a total of 4271 leaves from ten deciduous and two evergreen tree species. The authors demonstrate that leaf fresh mass scales more strongly with leaf area and that deciduous species tend to invest less biomass per unit leaf light-harvesting area than evergreen species. Another paper on leaf allometry by Jiao et al. examines if lamina mass vs. area scaling relationships are influenced by leaf age. They measured the leaf functional traits of five leaf-age groups of the evergreen broad-leaves shrub *Photinia × fraseri* using a total of 1,736 leaves, and observed that leaf area, and the ratio of lamina dry mass to lamina fresh mass increases with increasing leaf age. Their data indicate that leaves undergo a transition from resource acquisition to resource conservation during their development and growth.

## Leaf economics spectrum: trait relationships and ecological implications through environmental gradients

The leaf economics spectrum includes two easily-assessed traits, specific leaf area (SLA) and leaf dry matter content (LDMC), that scale with the plant resource harvesting strategy of the leaf economics spectrum (Wright et al., 2004). Fast-return species have high SLA and low LDMC, whereas slow-return species have the opposite combination of these traits (Wright et al., 2004). Thus, efforts to characterize the variation in these traits over large scales are very useful to map the functional characteristics of vegetation. Wang et al. investigated the effects of climatic and soil factors on plant resource utilization strategies using data collected from 926 plots across 163 forests in China and confirm the presence of significant differences in SLA and LDMC among plant functional types. In addition, they show that SLA decreases with increasing temperature and decreasing rainfall amount, and vegetation growing in these conditions exhibit conservative resource utilization. These findings are useful for predicting the effect of increasing global temperatures on plant resource utilization in the studied ecosystems.

The functional traits of leaves and the traits of other plant organs change in a correlated manner because the plant body is an integrated phenotype. In addition, annual and perennial species often manifest different traits. In general, annual species are characterized by a more rapid resource acquisition strategy compared to perennials (e.g., short leaf life-spans, higher specific leaf area, and leaf nutrient concentrations) (Lusk, 2019; Niinemets, 2020). However, there are fewer comparisons among root traits between annuals and perennials (Roumet et al., 2006), particularly in arid and semi-arid areas. Ning et al. examined 12 leaf and root traits of 54 dominant species from Northeastern China and report, among other differences, that annuals have higher individual leaf area and specific root length, but lower leaf dry matter content, leaf tissue density, and leaf and fine root dry matter content compared to perennials. Their findings indicate that annuals and perennials are characterized by distinct suites of leaf and root traits, and biomass allocation strategies, leading to differences in resource acquisition. These differences collectively provide an explanation for variation in species adaptability to water limitations in dry grasslands.

The large-scale effects of global climate change on grassland productivity remain poorly understood, and studies of leaf traits can provide important insights into how vegetation may likely respond to global changes. Using 182 grassland samples established in 17 alpine meadows (AM) and 21 desert steppes (DS) in China, Wang et al. show that the net primary productivity (NPP) of alpine meadows is higher than that of desert steppes and that NPP increases with increasing leaf nitrogen content and leaf phosphorus content, but decreases with increasing leaf dry matter content. These and other findings provide additional insights for predicting the effects of global climate change on the NPP of grasslands.

Elevation is one of the driving factors leading to leaf trait variation (Midolo et al., 2019). Yang et al. measured six leaf traits of 257 woody species at 26 elevations ranging from 1,050 to 3,500 m within the Tibetan Plateau and analyzed the scaling relationships among leaf fresh and dry mass, and area. Their analyses indicate that plants respond to differences in elevation by changing leaf area and biomass investment and coordinating scaling relationships among traits, although leaf trait variation along the elevation gradient had a minor effect on the numerical values of scaling exponent. In two other studies of the effects of altitude on plant functioning, Zhang et al. and Li et al. looked at plant intraspecific trait variation (ITV) along elevational gradients. Zhang et al. compared the leaf stoichiometry of the Northern hemisphere generalist perennial species *Potentilla anserina* growing at different elevations in the Middle-Eastern part of Qilian Mountains. With an increase in elevation, leaf carbon concentrations were observed to decrease, whereas leaf nitrogen concentration and leaf nitrogen to phosphorous concentration ratio increase, indicating that phosphorus imposes a stronger limitation of growth on *P. anserina* at higher elevations. Overall, this study confirms that plants acclimate to changes in elevation by altering the stoichiometry of their leaves to enhance carbon gain during shorter and cooler growing seasons. However, in this specific setting, acclimation to shorter and cooler growing conditions was limited by phosphorus availability. Li et al. studied ITV in the endangered dioecious *Taxus fuana* in small isolated populations endemic to the Himalayas region. They examined 18 leaf traits from 179 ovulate and pollen bearing trees along an elevational gradient in Gyirong County, Tibet, China, and they assessed ITV and sources of variation in leaf traits. Pollen bearing (“male”) plants were more tolerant to the environmental stresses at higher elevation, whereas the leaf traits of ovulate (“female”) plants were more responsive to elevation. However, the stronger plasticity of “females” was not associated with improved fitness. Unfortunately, these differences are likely to be detrimental to the maintenance of *T. fuana* populations.

The papers in this section significantly enlarge our understanding of leaf trait correlations, trait correlations across plants, and highlight multiple opportunities to study how leaf traits can be used to understand how plants interact with the environment and current and future climates.

## Role of leaf traits in environmental stress responses

Plants have evolved an array of mechanisms to deal with environmental stress. Among different environmental stresses, water availability is a key environmental factor affecting plant species distributions. However, the adaptive responses of congeneric species among sites differing in soil water availabilities remains unclear. Zhang et al. examined leaf economics and stem hydraulic traits in two *Quercus wutaishanica* dominated forests, a humid site in Qinling Mountains and a dry site in Loess Plateau, asking whether congeneric species have different economic and hydraulic traits across regions. They observed greater hydraulic safety and a stronger coordination of leaf economics and stem hydraulic traits in the dry site, and a greater hydraulic efficiency in the humid site. These results demonstrate that congeneric species utilize different types of adaptation mechanisms to maximize fitness in environments with different water availability. To further extend our understanding of the role of traits explaining species success along water availability gradients, Wang and Wen established two transects in the grasslands of Losses Plateau (LP) and Inner Mongolia Plateau (MP) to examine the distribution of intrinsic leaf water use efficiency (i.e., the ratio of net assimilation rate per unit stomatal conductance to water vapor) in coexisting species along aridity gradients. Intrinsic water use efficiency (iWUE) is a critical ecophysiological trait that characterizes the capacity of plants to cope with water- and nutrient-limited habitats in arid and semi-arid regions. The relationships between iWUE and a multi-dimensional functional trait spectrum indicated that species have evolved species-specific strategies to adapt to aridity by partitioning limiting resources. Thus far, the ranges of variation of iWUE in coexisting species along aridity gradients and the factors controlling the magnitude of interspecific variation have been poorly known. These findings highlight the interactive effects of limiting resources and leaf functional traits on plant adaptation strategies exploited to enhance iWUE.

In this Research Topic, Luo et al. have taken a biochemical approach to gain insight into acclimation responses to excess and limited water availabilities. In particular, they examined the time-dependent changes in ascorbate (AsA) and glutathione (GSH) contents, and the activities of enzymes involved in the AsA-GSH cycle in the perennial grass *Deschampsia caespitosa* in response to waterlogging and drought stresses. The authors reported that, in general, the activity of the AsA-GSH metabolic pathway increased with increasing both waterlogging and drought stress severity to reduce oxidative stress. Their findings provide information of biochemical responses of *D. caespitosa* to changes in water regimes, and as such constitute an important step for accelerating ecological restoration of degradation alpine marshes in the Qinghai-Tibetan Plateau.

Urbanization affects a range of morphological and physiological plant traits. However, plants can adopt different strategies to acclimate to urbanization pressures (Calfapietra et al., 2015). Xiao et al. examined the physiological and photosynthetic properties and heavy metal concentrations of four different plant functional groups (i.e., herbs, shrubs, subcanopy trees, and canopy trees, with eight species in total) located in urban, suburban, and rural areas. The authors report that canopy and subcanopy species acclimated to urbanization by reducing resource acquisition, but improving defense capacity, whereas the herb and shrub species improve resource acquisition as an acclimation response to urbanization. The evidence that different plant functional groups respond differently to urbanization is a valuable addition to our understanding plant adaptability to highly stressful urban environments and of high practical value for urban forestry.

The plant stress papers in this Research Topic jointly indicate that leaf traits are highly informative for assessing the severity of stress, and the degree of and capacity for stress acclimation.

## Application of leaf traits to understand patterns in the past

Finally, we turn to using leaf functional traits to inform us about the distant past. The Chicxulub bolide impact has been linked to a mass extinction of plants at the Cretaceous—Paleogene boundary (KPB) approximately 66 Mya, but how this event affected the evolution of plant ecological strategies has been understudied. Butrim et al. studied 1303 fossil leaves from KPB sediments in the Denver Basin, Colorado. Using a leaf allometry-based method to estimate leaf dry mass per area (LMA), they found no evidence for a shift in LMA across the KPB. However, in the Denver Basin, local environmental conditions appear to have played a larger role in determining viable leaf-economic strategies than any potential global signal associated with the Chicxulub bolide impact.

## Outlook

This collection of papers covers a broad range of topics that nevertheless all focus on the central question of how leaf functional traits inform our understanding of plant performance at different levels of biological organization. This collection emphasizes that we have only begun to fully uncover the many complex interrelationships among leaf traits, and their relationships to the traits of other plant organs, particularly roots. This suite of papers also highlights a number of broad and unique variation patterns in leaf traits in response to environmental limitations across gradients of water availability and along elevational gradients. It also demonstrates that different combinations of traits determine plant adaptability to specific environmental conditions. Although broad-scale variation patterns among key leaf traits have been identified in the past, the papers in this collection demonstrate that it is highly relevant to analyze the trait relationships in specific environmental contexts to draw inferences of plant performance, and to accurately predict changes in future vegetation. We hope that this Research Topic inspires future research in this challenging but vital and fruitful field of study.

## Author contributions

KN conceived and produced the first draft. ÜN reorganized, revised, and extended the subsequent version of the MS, and PS, JG, and JS edited the final draft. All authors contributed to the article and approved the submitted version.
